# Navigating Neural Landscapes: A Comprehensive Review of Magnetic Resonance Imaging (MRI) and Magnetic Resonance Spectroscopy (MRS) Applications in Epilepsy

**DOI:** 10.7759/cureus.56927

**Published:** 2024-03-25

**Authors:** Prasad Desale, Rajasbala Dhande, Pratapsingh Parihar, Devyansh Nimodia, Paritosh N Bhangale, Dhanajay Shinde

**Affiliations:** 1 Radiodiagnosis, Jawaharlal Nehru Medical College, Datta Meghe Institute of Higher Education and Research, Wardha, IND

**Keywords:** personalized medicine, advanced imaging techniques, magnetic resonance spectroscopy (mrs), magnetic resonance imaging (mri), neuroimaging, epilepsy

## Abstract

This review comprehensively explores the evolving role of neuroimaging, specifically magnetic resonance imaging (MRI) and magnetic resonance spectroscopy (MRS), in epilepsy research and clinical practice. Beginning with a concise overview of epilepsy, the discussion emphasizes the crucial importance of neuroimaging in diagnosing and managing this complex neurological disorder. The review delves into the applications of advanced MRI techniques, including high-field MRI, resting-state fMRI, and connectomics, highlighting their impact on refining our understanding of epilepsy’s structural and functional dimensions. Additionally, it examines the integration of machine learning in the analysis of intricate neuroimaging data. Moving to the clinical domain, the review outlines the utility of neuroimaging in pre-surgical evaluations and the monitoring of treatment responses and disease progression. Despite significant strides, challenges and limitations are discussed in the routine clinical incorporation of neuroimaging. The review explores promising developments in MRI and MRS technology, potential advancements in imaging biomarkers, and the implications for personalized medicine in epilepsy management. The conclusion underscores the transformative potential of neuroimaging and advocates for continued exploration, collaboration, and technological innovation to propel the field toward a future where tailored, effective interventions improve outcomes for individuals with epilepsy.

## Introduction and background

Epilepsy, a neurological disorder characterized by recurrent seizures, affects millions of individuals worldwide, posing significant challenges to both patients and healthcare providers. This review aims to comprehensively explore the role of magnetic resonance imaging (MRI) and magnetic resonance spectroscopy (MRS) in the context of epilepsy research and clinical practice [1]. Epilepsy encompasses a diverse range of disorders marked by abnormal electrical activity in the brain, leading to seizures. These seizures can manifest in various forms, from momentary lapses of awareness to convulsions. The multifaceted nature of epilepsy poses diagnostic complexities, making it imperative to delve into advanced neuroimaging techniques for a thorough understanding of the underlying neural dynamics [2].

Neuroimaging has emerged as a pivotal tool in the diagnosis and management of epilepsy. Traditional methods often fail to capture subtle structural and functional abnormalities within the brain that may contribute to seizure activity. With its non-invasive nature and high spatial resolution, MRI has revolutionized the field by enabling detailed visualization of the brain’s anatomy. This capability proves crucial in identifying lesions, malformations, and other structural anomalies that may be associated with epilepsy [3]. In addition to structural imaging, functional neuroimaging techniques, such as functional MRI (fMRI), provide insights into the dynamic aspects of brain function. Understanding how different brain regions interact during seizures or in the interictal period enhances our comprehension of the epileptic network [4].

The primary goal of this review is to navigate the intricate landscape of neural imaging, focusing on the applications of both MRI and MRS in the realm of epilepsy. MRI, with its ability to offer detailed anatomical information, and MRS, providing biochemical insights into brain metabolism, present a powerful combination for unraveling the complexities of epileptic pathophysiology. By critically examining the current state of MRI and MRS applications, we aim to understand their utility in epilepsy research and clinical settings comprehensively. From detecting subtle structural changes to probing alterations in neurotransmitter levels, these imaging modalities offer valuable tools for refining diagnosis, treatment planning, and monitoring disease progression.

## Review

Magnetic resonance spectroscopy in epilepsy

Overview of Magnetic Resonance Spectroscopy

MRS is a formidable complement to traditional MRI, providing a distinctive lens into the chemical composition of tissues in a non-invasive fashion. Diverging from conventional imaging methods, MRS delves into the realm of molecular insights, explicitly targeting the detection and quantification of various metabolites within the intricate landscape of the brain [5].

Unlike the anatomical focus of MRI, which primarily captures detailed images of tissues and structures, MRS extends its reach to the molecular level. By harnessing the principles of nuclear magnetic resonance, MRS examines the resonance frequencies of specific atomic nuclei present in metabolites, offering a unique spectroscopic signature for each compound. This nuanced approach identifies and measures various molecules, including neurotransmitters, amino acids, and energy-related metabolites [6].

The non-invasive nature of MRS is a hallmark of its utility, allowing for repeated measurements without exposing patients to ionizing radiation or invasive procedures. As a result, MRS stands as a powerful tool in both research and clinical settings, providing valuable insights into the biochemical intricacies of the brain. From investigating altered metabolic profiles in neurological disorders to guiding treatment strategies, MRS contributes to a comprehensive understanding of brain function and pathology, expanding the horizons of neuroimaging capabilities [7].

Principles of Magnetic Resonance Spectroscopy in Measuring Brain Metabolites

At its core, MRS relies on the principles of nuclear magnetic resonance, capturing the resonant frequencies of specific atomic nuclei within metabolites. Protons, phosphorous, and carbon-13 are among the nuclei commonly targeted, allowing for the measurement of various metabolites such as N-acetyl aspartate (NAA), creatine, choline, and gamma-aminobutyric acid (GABA). By analyzing the concentrations of these metabolites, MRS provides a window into the biochemical milieu of the brain [8]. Understanding the principles of MRS is essential for interpreting the significance of alterations in metabolite levels, which can serve as potential biomarkers for epileptic activity and its associated pathophysiological changes [9].

Applications of Magnetic Resonance Spectroscopy in Epilepsy Research

Detection of neurochemical changes in epileptic foci: MRS is pivotal in identifying neurochemical alterations associated with epileptic foci. Notably, changes in metabolite concentrations, particularly reductions in NAA, often indicate neuronal damage or dysfunction. The ability of MRS to pinpoint these alterations contributes significantly to localizing and characterizing epileptic zones within the brain. This not only enhances our understanding of the focal nature of epilepsy but also plays a crucial role in refining surgical planning for individuals with epilepsy. By providing insights into the specific neurochemical changes occurring in epileptic foci, MRS is an invaluable tool in unraveling the complexities of this neurological disorder [10].

Quantification of neurotransmitters: One of the distinctive capabilities of MRS lies in its ability to quantify neurotransmitters, presenting a unique opportunity to delve into the neurochemical basis of epilepsy. The measurement of neurotransmitters, including GABA and glutamate, allows researchers to explore the intricate balance between neuronal excitation and inhibition. GABA, the primary inhibitory neurotransmitter, and glutamate, the principal excitatory neurotransmitter, play critical roles in modulating neuronal activity. MRS, by enabling the measurement of changes in these neurotransmitters, offers valuable insights into the underlying mechanisms of epileptic activity. This aspect of MRS contributes significantly to advancing our understanding of the neurochemical intricacies that drive epilepsy [11].

Evaluation of treatment response: MRS emerges as a dynamic tool for monitoring treatment response in individuals with epilepsy. The changes in metabolite levels observed post-treatment indicate alterations in neuronal health and function. The ability to assess these changes over time provides clinicians with valuable information regarding the efficacy of therapeutic interventions. Furthermore, MRS plays a crucial role in elucidating the neurochemical basis of treatment response, shedding light on the specific biochemical changes associated with successful intervention. This not only aids in refining treatment plans but also contributes to the ongoing development of more targeted and personalized therapeutic approaches in epilepsy management. The dynamic insights offered by MRS enhance the precision and effectiveness of therapeutic strategies, ultimately aiming for improved outcomes for patients with epilepsy [12].

Integrated neuroimaging approaches

Combining Magnetic Resonance Imaging and Magnetic Resonance Spectroscopy for Comprehensive Assessment

The synergistic integration of MRI and MRS presents a robust and comprehensive approach to neuroimaging, offering a dual perspective on the brain’s structural and biochemical dimensions. This collaborative framework enables a more nuanced understanding of brain health and pathology, enriching the diagnostic and research potential of neuroimaging [13]. The structural information provided by MRI, including identifying lesions or abnormalities in brain anatomy, lays a foundational understanding of the spatial context of neurological conditions. However, the holistic assessment is achieved by complementing this structural insight with the biochemical details offered by MRS. For instance, while an MRI may reveal a structural abnormality in a specific brain region, MRS can delve into the neurochemical changes associated with that anomaly. This integration facilitates a more complete and intricate understanding of the pathophysiological alterations occurring within that specific area, transcending the limitations of a singular modality [14]. The collaborative application of MRI and MRS enhances diagnostic accuracy and contributes to a deeper comprehension of the underlying mechanisms driving neurological disorders. This integrated approach unlocks a wealth of information by bridging the structural and biochemical realms, fostering a more comprehensive assessment essential for advancing research and clinical practices in neurology and neuroimaging.

Case Studies Illustrating the Synergistic Use of Magnetic Resonance Imaging and Magnetic Resonance Spectroscopy in Epilepsy Diagnosis

MRI and MRS are valuable tools in diagnosing and managing epilepsy. MRI can identify structural abnormalities in the brain that may be causing seizures, guiding treatment and prognosis. It is recommended for all patients with epilepsy, as it can reveal structural abnormalities in a high percentage of cases, especially those with recurrent seizures. On the other hand, MRS is used as an adjunct diagnostic technique to identify the seizure focus, and it may reveal metabolic abnormalities that are more widespread than those seen on MRI. MRS can also help determine optimal drug regimens and study epileptogenic networks in the brain [15-19]. Single-voxel and multivoxel 1H-MRS have high sensitivity for detecting low NAA, indicative of neuronal dysfunction in focal epilepsies [17].

Advantages and Limitations of Integrated Neuroimaging Approaches

Understanding the advantages and limitations of integrated neuroimaging approaches is paramount for their effective utilization in epilepsy research and clinical practice, as described in Table 1 [20].

**Table 1:** Advantages and limitations of the integrated neuroimaging approaches. MRI = magnetic resonance imaging; MRS = magnetic resonance spectroscopy

Advantages	Limitations
Thorough understanding: Integration of structural and biochemical information provides a holistic understanding of epilepsy, encompassing both anatomical abnormalities and neurochemical alterations. This comprehensive insight can enhance diagnostic accuracy and treatment planning	Technical challenges: Integrating multiple neuroimaging modalities requires specialized equipment and expertise in MRI and MRS. Setting up and maintaining such systems can be resource-intensive, posing challenges in terms of equipment availability and technical support
Improved localization: Combined MRI and MRS data can enhance the precision of localizing epileptic foci within the brain. Accurate localization is crucial for surgical planning, minimizing the risk of damage to healthy brain tissue during surgical interventions	Complex analysis: Analyzing integrated neuroimaging data is inherently complex, requiring advanced skills in neuroimaging analysis. Interpreting combined datasets necessitates sophisticated algorithms and expertise, which may not be readily available in all clinical settings
Personalized treatment strategies: Integrated neuroimaging enables the development of personalized treatment plans based on individual structural and neurochemical profiles. Tailoring treatment strategies to specific patient characteristics can optimize therapeutic outcomes and minimize adverse effects	Cost and accessibility: Integrating multiple imaging modalities can increase the overall cost of neuroimaging studies. Moreover, access to advanced imaging techniques such as MRI and MRS may be limited in certain healthcare settings, hindering widespread adoption in clinical practice
Longitudinal monitoring: Integrated neuroimaging facilitates longitudinal monitoring of epileptic conditions, allowing clinicians to track changes in brain structure and neurochemistry over time. This longitudinal assessment can provide valuable insights into disease progression and treatment efficacy	Data integration challenges: Combining data from different imaging modalities requires robust methods for data integration and fusion. Ensuring compatibility and consistency between datasets obtained from MRI, MRS, and other modalities can be challenging, potentially leading to errors in analysis and interpretation
Research advancements: Integrated neuroimaging approaches drive advancements in epilepsy research, enabling researchers to investigate complex relationships between brain structure, neurochemistry, and epileptogenesis. These approaches contribute to the development of novel diagnostic tools and therapeutic interventions	Interpretation variability: Integrating data from multiple neuroimaging modalities may lead to variability in interpretation due to differences in imaging protocols, data preprocessing techniques, and analytical methodologies. Standardizing procedures and protocols across imaging centers is essential to mitigate interpretation variability and ensure consistency in results

Advanced magnetic resonance imaging techniques in epilepsy

High-Field Magnetic Resonance Imaging and Its Impact on Epilepsy Imaging

In recent years, high-field MRI has significantly elevated the precision and depth of imaging in epilepsy research and clinical applications. High-field MRI systems, operating at 3 Tesla (T) or higher, provide superior signal-to-noise ratios and enhanced spatial resolution compared to conventional lower-field systems. This heightened sensitivity detects subtle structural abnormalities that might be overlooked in standard MRI [21]. The impact of high-field MRI in epilepsy imaging extends beyond anatomical visualization. It enables more accurate localization of epileptic foci, aiding in identifying minute lesions or abnormalities that may be crucial for surgical planning. The increased clarity of imaging provided by high-field MRI contributes to a more comprehensive understanding of the structural intricacies underlying epilepsy, ultimately refining diagnostic accuracy and therapeutic interventions [21].

Emerging Techniques Such as Resting-State Functional Magnetic Resonance Imaging and Connectomics

Beyond structural imaging, emerging techniques such as resting-state fMRI and connectomics have emerged as valuable tools for unraveling the functional connectivity patterns in the brain affected by epilepsy. Resting-state fMRI allows researchers and clinicians to examine spontaneous fluctuations in blood oxygen level-dependent signals during rest periods, providing insights into the brain’s intrinsic functional architecture [22].

Connectomics, a network-based approach, involves mapping and analyzing the complex connections between different brain regions. In the context of epilepsy, these techniques offer a holistic view of altered connectivity patterns, shedding light on how aberrant networks may contribute to seizure generation and propagation. Integrating structural and functional connectomics further refines our understanding of the broader neural networks implicated in epilepsy [23].

The Role of Machine Learning in Analyzing Complex Neuroimaging Data

As neuroimaging datasets grow in complexity and size, machine learning (ML) becomes increasingly pivotal in extracting meaningful information. ML algorithms demonstrate remarkable capabilities in analyzing intricate neuroimaging data, offering new avenues for understanding, predicting, and classifying epileptic phenomena [24]. ML applications in epilepsy neuroimaging encompass various tasks, including automated detection of epileptic lesions, prediction of treatment outcomes, and classification of different epilepsy subtypes based on imaging features. Integrating ML techniques with advanced MRI data facilitates the development of predictive models, aiding clinicians in personalized treatment planning and decision-making [25].

Clinical applications and challenges

Use of Neuroimaging in Pre-surgical Evaluation

Neuroimaging plays a pivotal role in the pre-surgical evaluation of individuals with epilepsy, especially those who are candidates for surgical intervention. High-resolution structural MRI assists in precisely localizing epileptic foci and identifying the anatomical structures involved. This information is crucial for neurosurgeons in planning and executing precise surgical procedures, such as resective surgery or neuromodulation, to control or alleviate seizures [26]. Advanced imaging techniques, including fMRI and diffusion tensor imaging, contribute to the delineation of eloquent brain regions and white matter tracts critical for cognitive and motor functions. Integration of functional and structural data enhances the precision of surgical planning, minimizing the risk of postoperative deficits [27].

Monitoring Treatment Response and Disease Progression

Longitudinal neuroimaging is a valuable tool for monitoring treatment response and understanding the progression of epilepsy over time. Serial MRI scans allow clinicians to assess structural changes, track lesions’ evolution, and evaluate therapeutic interventions’ impact. Functional imaging techniques, such as fMRI and positron emission tomography, contribute insights into changes in brain activity associated with treatment response [28]. MRS plays a crucial role in monitoring biochemical alterations in the brain, providing a dynamic perspective on the effects of antiepileptic medications and other therapeutic interventions. These monitoring strategies aid in refining treatment plans, adjusting medication regimens, and exploring alternative therapies based on individual patient responses [29].

Challenges and Limitations in Incorporating Neuroimaging Into Routine Clinical Practice

Cost and accessibility: High-field MRI and advanced neuroimaging techniques, while offering unparalleled insights, present a considerable financial barrier, limiting accessibility for specific patient populations and healthcare facilities. The initial capital investment, maintenance costs, and the need for specialized staff contribute to the overall expense. This financial constraint may disproportionately affect underserved communities and smaller healthcare institutions, hindering their ability to provide patients with the benefits of state-of-the-art neuroimaging [30].

Interpretation complexity: The interpretation of neuroimaging data is a highly specialized task that demands expertise in the technical aspects of imaging and the clinical intricacies of neurological conditions. As technology evolves, the complexity of interpreting integrated data from multiple modalities increases. Ongoing training is essential for clinicians to stay abreast of evolving technologies and methodologies, ensuring accurate and meaningful interpretation. This demand for continuous education strains healthcare systems and requires a commitment to professional development among neuroimaging practitioners [31].

Standardization and reproducibility: Achieving standardization in neuroimaging protocols across different institutions remains a formidable challenge. The lack of uniformity can impact the reproducibility and comparability of results, hindering the progress of collaborative research efforts. Ongoing efforts to establish imaging protocols and quality assurance measures are essential to mitigate these challenges. Collaborative initiatives, interdisciplinary communication, and the development of standardized guidelines are crucial for advancing the field and ensuring the reliability of neuroimaging findings across diverse clinical and research settings [32].

Limited temporal resolution: Despite the remarkable capabilities of structural MRI, some imaging modalities may need more temporal resolution to capture rapid changes in brain activity during seizures. This limitation poses challenges in precisely characterizing the dynamics of epileptic events, potentially impacting the accuracy of diagnosis and treatment planning. Innovations in imaging technology focused on improving temporal resolution are crucial for advancing our understanding of the temporal aspects of epilepsy, providing more precise insights into the mechanisms and evolution of seizures [33].

Ethical considerations: Integrating advanced imaging technologies in epilepsy research and clinical practice brings forth a set of ethical considerations. Issues related to patient privacy, informed consent, and the responsible use of sensitive neuroimaging data require careful consideration. Striking a balance between advancing medical knowledge and safeguarding patient rights is imperative. Ethical frameworks and guidelines must evolve with technological advancements, ensuring that ethical standards are maintained throughout acquiring, storing, and utilizing neuroimaging data to benefit patients and scientific progress [34].

Future directions and innovations

Promising Developments in Magnetic Resonance Imaging and Magnetic Resonance Spectroscopy Technology

Ultra-high field MRI: Pushing the boundaries of MRI, pursuing ultra-high-field MRI, exceeding 7 T, represents a significant leap in imaging capabilities. This innovation holds immense promise by offering enhanced spatial resolution and sensitivity, potentially unlocking finer details of brain structures and abnormalities. In the context of epilepsy, ultra-high-field MRI stands as a beacon of hope for improved localization of epileptic foci. The heightened precision afforded by this technology holds the potential to refine pre-surgical planning, allowing neurosurgeons to navigate and intervene with unprecedented accuracy. As the magnetic field strength increases, so does the prospect of unraveling the subtle intricacies of the epileptic brain, contributing to advancements in diagnosis and tailored therapeutic interventions [35].

Quantitative MRI techniques: The advent of quantitative MRI techniques, exemplified by magnetic resonance fingerprinting and relaxometry mapping, signifies a paradigm shift in neuroimaging. These techniques offer a departure from traditional qualitative assessments, providing a more accurate and quantitative evaluation of tissue properties in the brain. In the realm of epilepsy research, quantitative MRI techniques contribute to a deeper understanding of subtle changes occurring within the brain, particularly those associated with epileptic conditions. The inherent precision and quantifiability in these methods hold the potential to unveil nuanced alterations, aiding researchers and clinicians in identifying and characterizing epileptic abnormalities with unprecedented accuracy. This advancement not only refines diagnostic capabilities but also paves the way for more targeted and personalized treatment strategies [36].

Real-time imaging: Advances in real-time MRI techniques usher in a new era of dynamic imaging with higher temporal resolution. This innovation holds particular significance in studying brain function, enabling the capture of rapid changes in activity, especially during seizures. Real-time imaging stands poised to illuminate the dynamics of epileptic networks, providing insights into the temporal evolution of seizures. By offering a glimpse into the intricate interplay of neural processes in real time, this technology has the potential to revolutionize our understanding of epilepsy’s temporal aspects. The ability to visualize and analyze brain activity moment-to-moment enhances our scientific understanding. It holds promise for more precise clinical interventions and personalized treatment strategies for individuals with epilepsy [37].

Potential Advancements in Imaging Biomarkers for Epilepsy

Metabolic imaging biomarkers: Exploring metabolic imaging biomarkers represents a promising frontier in epilepsy research. By expanding the application of MRS, researchers aim to identify specific metabolic signatures associated with different types of epilepsy. This innovative approach holds the potential to revolutionize the classification of epilepsy subtypes, offering a more nuanced understanding of the underlying metabolic dysregulations contributing to seizures. The identification of distinct metabolic profiles may not only enhance diagnostic precision but also guide the development of personalized treatment strategies. Metabolic imaging biomarkers can transform the landscape of epilepsy management by providing valuable insights into the unique biochemical fingerprints associated with diverse epileptic conditions [38].

Functional connectivity biomarkers: Advancements in resting-state fMRI and connectomics are poised to unveil specific patterns of altered functional connectivity that can serve as biomarkers for epilepsy. By probing the brain’s intrinsic functional architecture during rest periods, researchers seek to identify characteristic connectivity patterns associated with epileptic conditions. These functional connectivity biomarkers can facilitate early diagnosis and treatment optimization. The ability to discern distinct connectivity signatures associated with epilepsy enhances our understanding of the disorder and opens avenues for targeted interventions based on individual connectivity profiles. This innovation represents a significant stride toward personalized medicine in epilepsy, where treatment strategies can be tailored to address each patient’s specific functional connectivity alterations [39].

Quantitative structural biomarkers: The development of quantitative structural biomarkers derived from high-field MRI, such as cortical thickness and volumetric analysis, marks a critical advancement in tracking structural changes associated with epilepsy progression. These quantitative measures provide a more precise and objective assessment of alterations in brain structure over time. By serving as reliable biomarkers, they offer a means to monitor the evolution of structural abnormalities associated with epilepsy, aiding in research and clinical settings. Quantitative structural biomarkers can potentially revolutionize our approach to epilepsy by providing quantitative, data-driven insights into the progressive changes in cortical morphology. This development not only enhances our ability to understand the structural dynamics of epilepsy but also lays the foundation for more effective and targeted therapeutic interventions [40].

Implications for Personalized Medicine in Epilepsy Management

Tailored treatment strategies: The concept of tailored treatment strategies represents a significant advancement in integrating neuroimaging data into personalized medicine for epilepsy. By leveraging the insights provided by neuroimaging, clinicians can tailor treatment plans based on individual patient profiles. This may involve identifying the most effective medications, optimizing surgical approaches, or exploring neuromodulation techniques more precisely. The ability to customize treatment strategies based on the unique neuroimaging characteristics of each patient not only enhances therapeutic outcomes but also minimizes potential side effects. This approach marks a paradigm shift in epilepsy management, moving toward more individualized and targeted interventions considering the specific neuroanatomical and functional aspects revealed by advanced imaging modalities [41].

Predictive models: The development of predictive models using ML algorithms heralds a new era in utilizing neuroimaging data to forecast treatment responses and disease trajectories in epilepsy. By leveraging the vast and intricate information contained in neuroimaging datasets, these models have the potential to predict how an individual patient will respond to various treatments and how their condition may evolve. This capability aids in early intervention, allowing clinicians to tailor treatment plans proactively. Predictive models improve clinical decision-making and contribute to more favorable long-term outcomes by identifying patients’ most effective therapeutic approaches based on their unique neuroimaging profile [42].

Therapeutic monitoring: Real-time monitoring of treatment responses through advanced imaging techniques is a transformative approach in epilepsy management. Clinicians can adjust the treatment plans as the patient’s condition evolves by continuously evaluating how the brain responds to treatment. This dynamic therapeutic monitoring allows for a more responsive and personalized approach, ensuring that interventions are optimized in real time based on the patient’s neuroimaging-derived response. This innovation represents a proactive strategy in epilepsy care, enabling clinicians to fine-tune treatments according to the evolving neural dynamics observed through continuous monitoring, ultimately improving the precision and effectiveness of therapeutic interventions [43].

## Conclusions

This comprehensive review has illuminated the intricate landscape of neuroimaging in epilepsy, particularly emphasizing the transformative applications of MRI and MRS. The synthesis of critical findings underscores the integral role of neuroimaging in deepening our comprehension of epilepsy, ranging from identifying structural anomalies to exploring dynamic changes in brain function. The advent of advanced techniques, including high-field MRI, resting-state fMRI, connectomics, and ML, has emerged as a catalyst for innovation, enriching both epilepsy research and clinical practice. The potential impact of MRI and MRS on advancing patient care is substantial, offering a multi-dimensional understanding of the epileptic brain and facilitating personalized treatment strategies. However, the journey does not end here; there is a resounding call to action for continued exploration and development in neuroimaging in epilepsy. This entails standardizing protocols, fostering interdisciplinary collaboration, prioritizing patient-centric research, and investing in technological advancements. Through these concerted efforts, we can aspire to a future where neuroimaging not only refines diagnostics and treatment planning but also contributes to a paradigm shift in how we approach and manage epilepsy, ultimately improving the quality of life for those affected by this neurological challenge.
